# Prenatal PPARα activation by clofibrate increases subcutaneous fat browning in male C57BL/6J mice fed a high-fat diet during adulthood

**DOI:** 10.1371/journal.pone.0187507

**Published:** 2017-11-02

**Authors:** Szu-Han Chen, Pei-Min Chao

**Affiliations:** Institute of Nutrition, China Medical University, Taichung, Taiwan; Vall d'Hebron Institut de Recerca, SPAIN

## Abstract

We tested the hypothesis that prenatal administration of PPARα agonist clofibrate may permanently increase browning capacity of developing white adipose tissue (WAT). Pregnant C57BL/6J mice were fed a basal diet, without (C) or with 0.5% clofibrate (CF, a PPARα agonist) throughout pregnancy. After parturition, only male offspring were used; all suckled their mothers (who were eating the C diet) and after weaning, they ate a standard chow diet for 4 wk, followed by a high-fat diet (HFD) for 5 wk. Administration of CF up-regulated serum concentrations and hepatic expression of FGF21 in fetuses, with a return to basal levels after CF withdrawal. At postnatal day 84 (P84), CF-offspring had significantly higher expression of thermogenic genes (*Ucp1*, *Cidea*, *Ppara Ppargc1a*, *Cpt1b*) and UCP1 protein levels in response to HFD in inguinal fat, but not in retroperitoneal (combined with perirenal) or epididymal fat. Based on UCP1 levels in inguinal fat on P7, P14, and P21, appearance of the transient brown-adipocyte phenotype seemed to be hastened by CF exposure. We concluded that giving CF to pregnant mice programmed greater HFD-induced WAT browning in subcutaneous, but not in visceral fat, in their male offspring at adulthood.

## Introduction

White and brown are 2 distinct adipose tissues for storage of excess energy and thermogenesis, respectively. In addition to traditional white and brown adipocytes, a third type of adipocyte, i.e. brown-in-white (brite) or beige cells, that emerge within white adipose tissue (WAT) are regarded as a plastic response to an energy surplus [1]. Beige/brite adipocytes are inducible, multilocular, UCP1-positive, and thermogenic (produce and dissipate heat) [2]. The relevance of this issue to anti-obesity was highlighted by ^18^ fluorodeoxyglucose-positron emission tomography detection of brown adipose tissue (BAT) in supraclavicular and neck regions of adult humans; furthermore, its activity was inversely correlated with BMI [3–5]. Human BAT had molecular signatures that resembled murine brite cells rather than classical brown adipocytes present in interscapular BAT [2]. Increased numbers of brite cells (by transgenic or pharmacological approaches) conferred health benefits, including resistance to diet-induced obesity (DIO) and amelioration of glucose intolerance, insulin resistance and liver steatosis [6].

In contrast to classical brown adipocytes with Myf5^+^ myocyte markers, brite cells are Myf5^-^, furthermore, conservation of the Hoxc9 marker indicates they have a shared lineage with white adipocytes [7]. Though brite cells are inducible by cold, β-adrenergic stimulation and PPAR agonists, it remains controversial whether their recruitment involves proliferation and differentiation of committed progenitors and/or transformation from a subset of white adipocytes with browning potentiality [8–10]. For the latter scenario, it is believed these brite cells have a white phenotype at thermoneutrality, but reversibly reveal their brown identity upon cold/β-adrenergic stimulation or consumption of a high-fat diet. In rodents, propensity to accumulate brite/beige cells is highly strain- and location-dependent [11, 12].

Fibroblast growth factor (FGF21) is a PPARα-regulated hepatokine involved in control of metabolism by modulating glucose homeostasis, insulin sensitivity and ketogenesis [13]. Its target tissues include BAT, WAT and brain expressing β-klotho, a co-receptor of FGF21 action [14]. The relevance of FGF21 in promoting WAT browning and thermogenesis was first postulated in transgenic mice and subsequently demonstrated in adipocyte culture; its mode of actions are more than endocrine, but also paracrine/autocrine [15–17]. Post-transcriptional increases in PGC1α protein, a critical regulator of mitochondria biogenesis and thermogenesis, contributed to FGF21-mediated UCP-1 induction in BAT and WAT [17]. Hondares et al. [18] demonstrated that during fetal-to-neonatal transition, milk suckling (a high-fat diet) upregulates hepatic *Fgf21* expression PPARα-dependently, thus switching-on BAT thermogenesis (which protects neonates from hypothermia). Accordingly, FGF21 is regarded as a thermogenic hormone [13, 18].

Treatment with PPAR agonists manipulates the differentiation program towards brite instead of white adipocytes in cell culture studies [7, 19, 20] and chronic administration of fibrate (PPARα activators) or thiazolidinedione (PPARγ activators) compounds induced WAT browning in mice and humans [21, 22]. Since most active adipogenesis *in vivo* occurs during the perinatal period [23], perhaps PPAR activation during this critical window would have a permanent effect on thermogenic capacity of WAT. We reported that ingestion of oxidized frying oil in pregnant C57BL/6J mice protected their male offspring from DIO in adulthood, along with higher expression of thermogenic genes in subcutaneous fat [24]. That frying oil contains PPARα activators [25, 26] and hepatic *Fgf21* expression in embryonic day 18 (E18) of frying oil-exposed fetuses was markedly elevated [24], we speculated that greater exposure to FGF21 during embryogenesis might direct adipogenesis toward brite within primitive WAT, thus conferring greater resistance to DIO. In this study, pregnant mice were given clofibrate (CF), a PPARα agonist which can cross the placenta [27], to up-regulate hepatic *Fgf21* expression in fetuses. A high-fat diet (HFD) is capable in inducing thermogenesis and UCP-1 up-regulation via sympathetic innervation not only in BAT, but also in WAT, presumably to dissipate excess energy and counteract weight gain in short time [28]. When offspring reached adulthood, they were fed a HFD to test DIO and serve as a moderate β-AR stimulator to reveal the brite phenotype.

## Materials and methods

### Animals and diets

**Fig 1:**
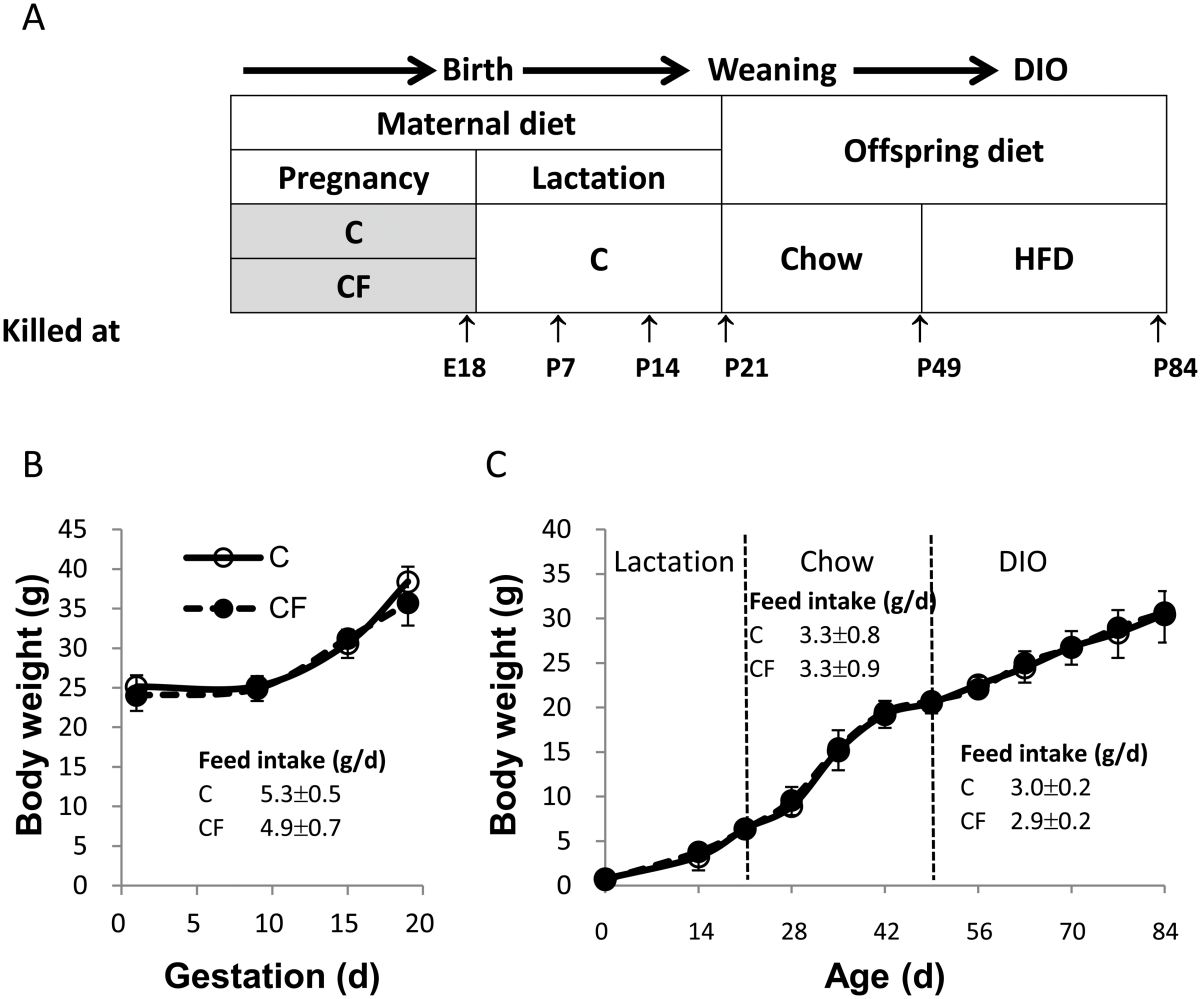
Study design (A), body weight of mothers during pregnancy (B) and of offspring from birth to 84 d of age (C). Feed intake is shown in inset tables of (B) and (C). Only male offspring were used in this study. Data are mean±SEM. For mothers, n = 5/group; for offspring, n = 22 (from 5 litters)/group.

Female and male C57BL/6JNarl mice purchased from the National Laboratory Animal Center of the National Applied Research Laboratories (Taipei, Taiwan) were used for breeding. Females with parity from 1 to 5 were used. The experimental protocol is shown (Fig 1A). Pregnant females were fed either a control (C) or experimental (CF) diet from breeding to parturition. The C diet was based on an AIN-93M diet [29] with a slight modification to contain 21 kcal% fat from soybean oil, whereas the CF diet was the C diet with addition of 0.5% clofibrate (Fluka, Buchs, Switzerland). Pregnancy was dated by the presence of a vaginal plug (defined as pregnancy day 1). After spontaneous parturition (pregnancy day 19.5±0.5), all littermates were uniformly nursed by dams fed the C diet for 3 wk, with litter sizes adjusted to 8–10, weaned onto a nonpurified standard diet (Altromin 1320, Lage, Germany) for 4 wk, and then switched to a HFD (51 kcal% fat, butter-based) for 5 wk. In this study, only male offspring were used and 2 groups of offspring were designated, according to their mother’s diet (C or CF). All mice were kept in a room maintained at 23±2°C, with a controlled 12-h-light:-dark cycle with *ad libitum* to feed and drinking water. Body weight and feed intake were recorded weekly. Protocols for animal care and handling were approved by the Institutional Animal Care and Use Committee of the China Medical University (104-130-N). Composition of all diets used are shown (S1 Table).

### Tissue sampling and biochemical analysis

Five mothers for each group were killed at pregnancy day 18 to collect blood, liver and fetuses (E18). Male offspring were killed at 7, 14, 21 and 84 d of age (P7, P14, P21 and P84) by carbon dioxide asphyxiation. Retroperitoneal combined with perirenal (RP+PR), inguinal (IG), epididymal (EP) fat pads and liver were excised and weighed. As it is problematic to separate RP and PR depots in obese mice, alternatively, RP and PR were combined for collection. Portions of WAT and liver were stored at −80°C for subsequent extraction of protein and RNA. Fetal, maternal, and post-natal serum was separated from whole blood by centrifugation (3000 x g for 15 min) and serum FGF21 concentrations were determined (rat/mouse FGF21 ELISA kit, Millipore, Darmstadt, Germany).

### Gender identification of fetuses

For accurate sexing of fetuses, the presence of the *Sry* and *DXNds3* genes was determined by PCR. The *Sry* gene is present in a particular sex-determining region of the Y chromosome, and *DXNds3* is a polymorphic microstatellite locus located on the mouse X chromosome [30]. Genomic DNA was extracted from fetal liver using FavorPrep Tissue genomic DNA extraction mini kits (Favorgen Biotech). The sequences of the primers used were 5-GCCATGTCAAGCGCCCCATGAA-3 (forward) and 5-GTGGGGATATCAACAGGCTGCCA-3 reverse) for Sry and 5-ATGCTTGGCCAGTGTACATAG-3 (forward) and 5- TCCGGAAAGCAGCCATTGGAGA-3 (reverse) for *DXNds3*. Fetuses with both *Sry* and *DXNds3* were identified as males.

### Immunoblotting

Samples of WAT were homogenized in a RIPA buffer containing 1% protease inhibitor cocktail and aliquots containing 50 mg of protein were electrophoresed on 10% SDS gels, transferred to a PVDF transfer membrane (Millipore, Darmstadt, Germany), and immunoblotted. Primary antibodies (diluted 1:1000 in PBS) were mouse antibodies against β-actin (Cat.#STJ31750; St John’s Laboratory, London, UK), tyrosine hydroxylase (TH) (Cat.#MAB318; Millipore, California) and rabbit antibodies against human uncoupling protein 1 (UCP1) (Cat.#ab10983; Abcam, Cambridge, UK). In addition, HRP-labeled goat anti-mouse IgG antibodies (Cat.#115-035-003; Jackson ImmunoResearch, West Grove, PA) and goat anti-rabbit IgG antibodies (Cat.#ab6721; Abcam) at a dilution of 1:5000 in PBS were used as a secondary antibody. Bound antibodies were detected using an enhanced chemiluminescence Western blotting kit (Amersham International, Uppsala, Sweden) and images quantified by densitometric analysis using a Multimage Light Cabinet (Alpha Innotech Corporation, San Leandro, CA).

### Immunohistochemical analysis

Inguinal fat was fixed in 10% formalin, dehydrated through a graded ethanol series, embedded in paraffin, and cut into 5 mm sections. After deparaffinization and rehydration, sections were incubated using 5% goat serum in PBS. The primary antibody used at a dilution of 1:100 in PBS was a rabbit antibody against human UCP1 (Abcam), whereas the secondary antibody was biotinylated goat anti-rabbit IgG antibodies (Dako, California) for UCP1 at a dilution of 1:250 in PBS. Sections for UCP1 staining were processed using a Dako kit (Dako REALTM envision TM detection system) according to the manufacturer’s instructions and examined on a BX60 microscope (Olympus, New York, NY).

### RNA isolation and mRNA detection

Total RNA was extracted from homogenized tissue using TRIZOL reagent (Invitrogen, California) according to the manufacturer’s instructions and 1 mg total RNA was reverse-transcribed into first-strand cDNA using 200 units of MMLV-RT (Promega, Wisconsin) in a total volume of 20 mL. For real-time PCR, a TaqMan system with inventory primers and probes (Applied Biosystems, California) or a SYBR system with self-designed primers was used. The assay ID of the inventory primers and probes and the sequence of the self-designed primers are shown (S2 Table). Amplification using 40 cycles of 2 steps (95°C for 15 s and 60°C for 1 min) was performed on an ABI Prism 7900HT sequence detection system.

### Statistical analysis

Data are expressed as mean ± SEM. Differences between groups at the same age were detected using a Student’s *t*-test. If variances were not homogeneous, data were log-transformed before statistical analysis. The SAS System (SAS Institute, Cary, NC, USA) was used for statistical analysis and differences considered significant at *P* < 0.05.

## Results

### Gestational CF on growth rate and adiposity of offspring

There was no difference in feed intake or body weight between C and CF-fed mothers throughout pregnancy (Fig 1B), nor in post-natal growth and daily feed intake of their offspring from 0 to 84 d of age (Fig 1C). The body fat percentage of RP+PR and IG did not differ between C and CF offspring at P84 (after DIO). However, the body fat percentage of EP was significantly lower in CF compared to C offspring (Table 1).

**Table 1:** Percentage of body fat from 3 depots of DIO-challenged adult males (at P84) from mothers receiving C or CF diet during pregnancy.

Body fat	C	CF
	% body weight	
Retroperitoneal+Perirenal	1.43±0.46	1.48±0.38
Epididymal	4.06±1.62	3.39±1.32*
Inguinal	2.91±0.78	2.82±0.52

Data are mean±SEM, n = 22 (from 5 litters)/group

*P<0.05.

### Gestational CF on PPARα activation of mothers and offspring

**Fig 2:**
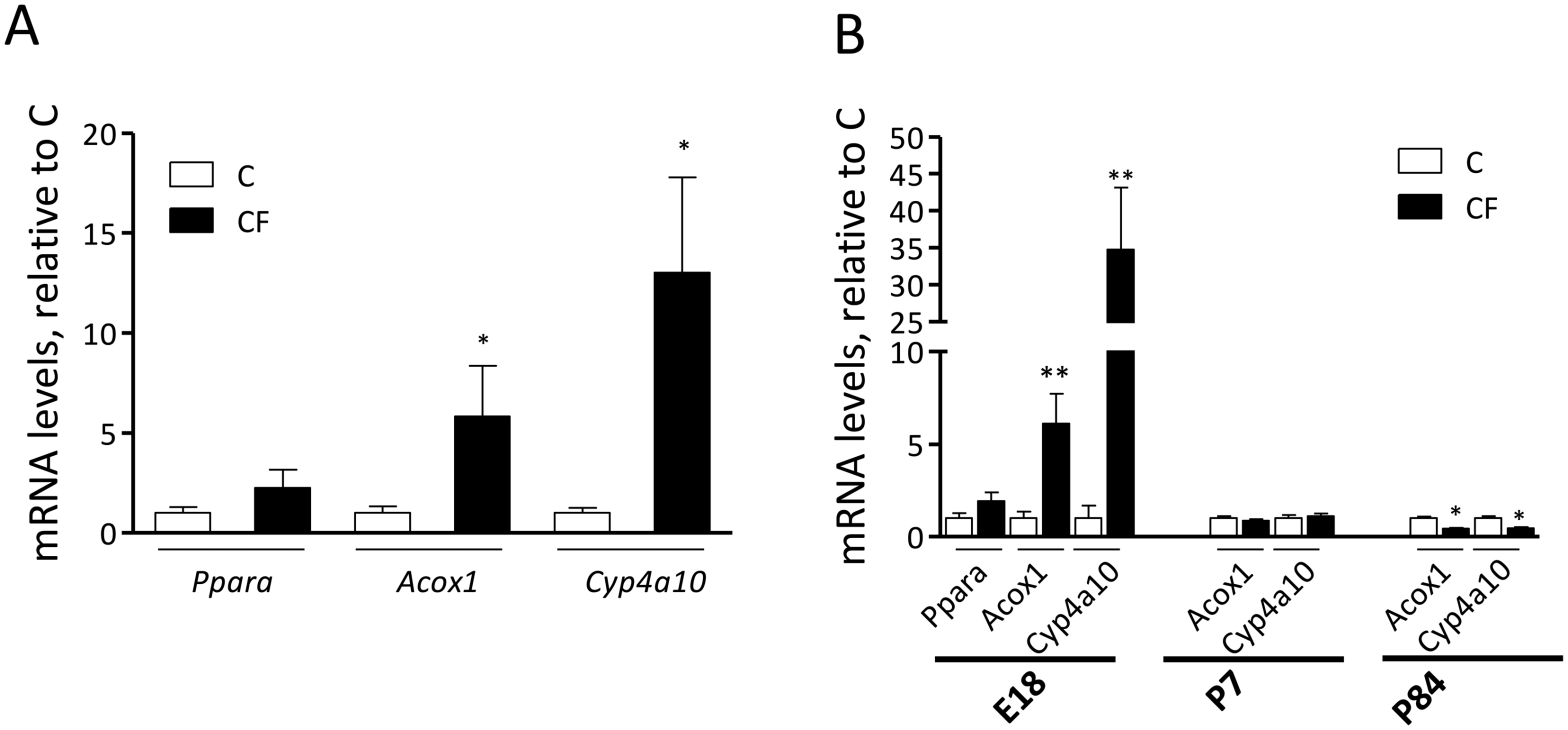
Hepatic mRNA levels of PPARα and its target genes of mothers at pregnancy day 18 (A) and their offspring at embryonic day 18 (E18), postnatal day 7 (P7) and day 84 (P84) (B). Data are mean±SEM. For mothers in (A), n = 5/group; for offspring in (B), n = 27 (from 5 litters)/group at E18, 12 (from 3 litters)/group at P7, 22 (from 5 litters)/group at P84. The comparison was based on mRNA levels relative to C (taken as 1) at the same time point. *P<0.05 and **P<0.01.

To confirm PPARα activation with CF treatment, hepatic mRNA levels of PPARα and its target genes were measured in mothers at pregnancy day 18 (Fig 2A) and offspring at E18, P7 and P84 (Fig 2B). In accordance with timing of CF administration, there were significantly higher transcripts of *Acox1* and *Cyp4a10* but no change in *Ppara* in CF-mothers and their offspring only at E18, but not during postnatal life, as compared to age-matched control peers. Maternal CF during gestation programmed offspring to have lower hepatic expression of *Acox* and *Cyp4a10* after DIO.

### Gestational CF on FGF21 of mothers and offspring

**Fig 3:**
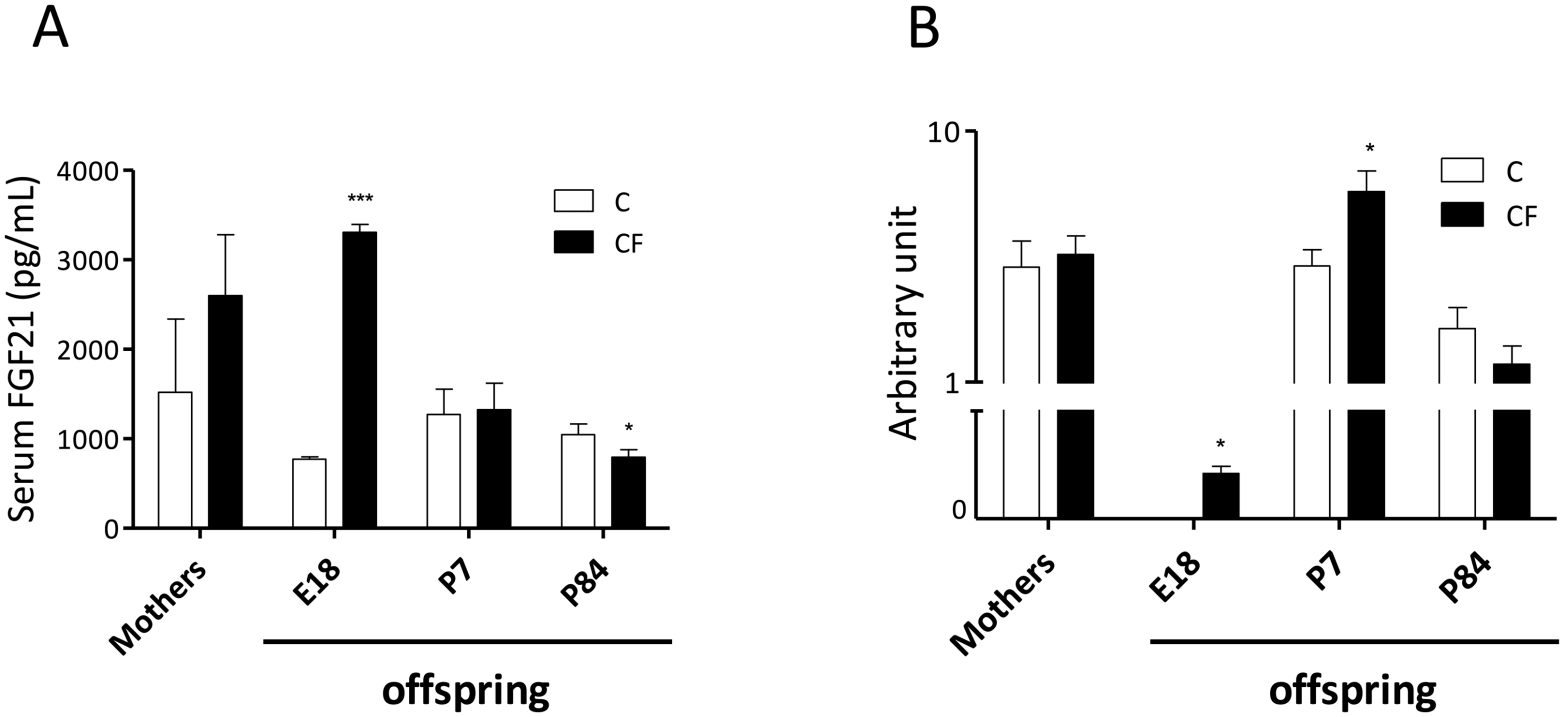
Serum concentrations (A) and hepatic mRNA levels (B) of FGF21 of mothers at pregnancy day 18, as well as their offspring at embryonic day 18 (E18), postnatal day 7 (P7) and day 84 (P84). Data are mean±SEM. For mothers, n = 5/group; for offspring, n = 27 (from 5 litters)/group at E18, 12 (from 3 litters)/group at P7, 22 (from 5 litters)/group at P84. *P<0.05 and ***P<0.005.

In pregnant mothers, CF caused a non-significant increase in serum FGF21 concentrations (*P* = 0.17; Fig 3A), whereas in offspring, CF significantly elevated serum FGF21 concentrations at E18, had no effect at P7, and reduced concentrations at P84. Generally, serum FGF21 is attributable to hepatic expression and secretion without cold stimulation [13]. Hepatic mRNA levels of *Fgf21* was not different between groups in mothers at pregnancy day 18, but was significantly higher in CF- relative to C- offspring at E18, sustained to P7, but not at P84 (Fig 3B).

### Gestational CF on WAT browning of adult offspring after DIO

**Fig 4:**
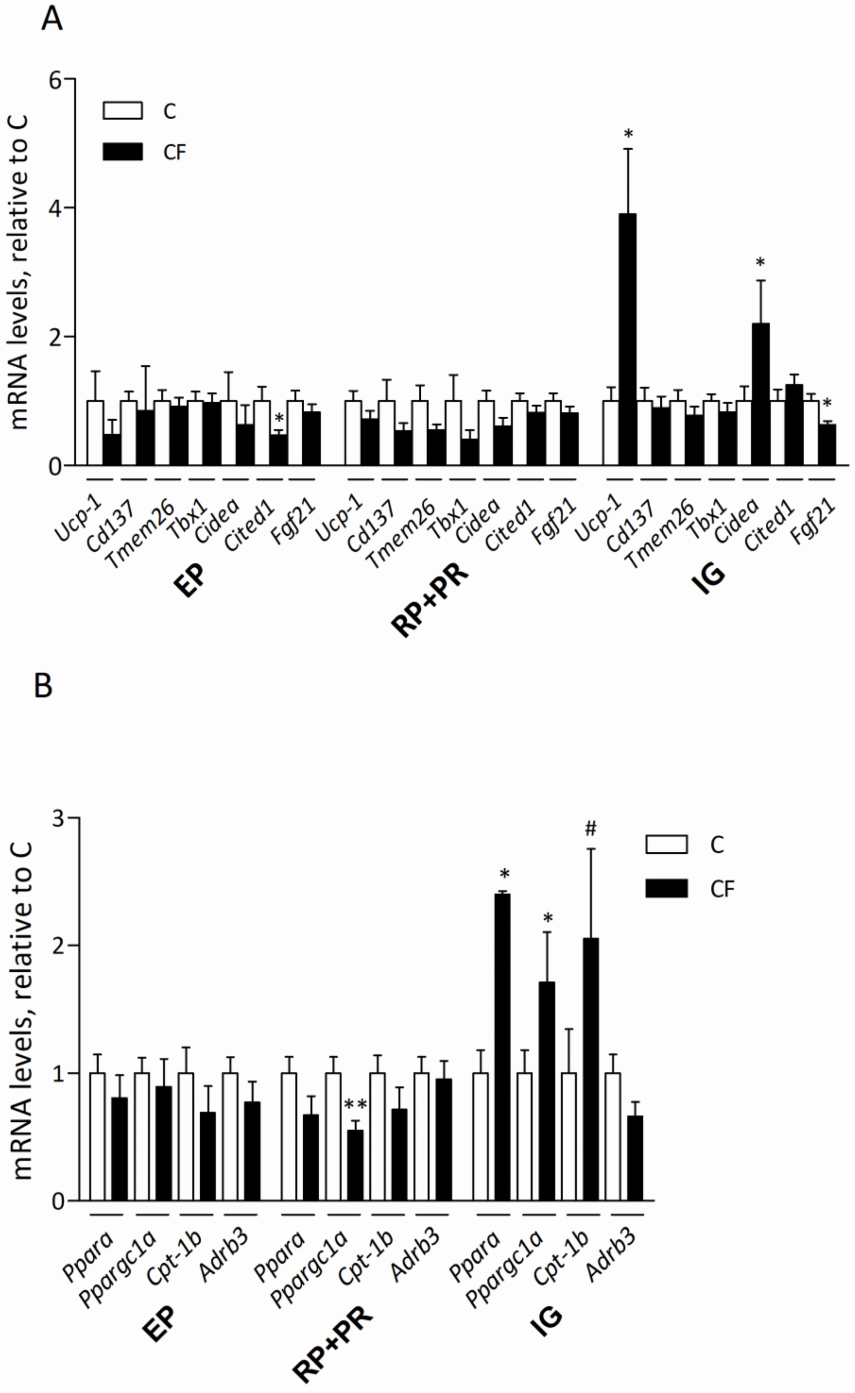
Gene expression of brown markers (A) and proteins associated with fatty acid β-oxidation (B) in 3 fat depots (EP, RP+PR and IG representing epididymal, retroperitoneal combined perirenal, and inguinal fat, respectively) of offspring at P84. Data are mean±SEM, n = 22 (from 5 litters)/group. The comparison was based on the mRNA levels relative to C (taken as 1) within the same fat depot. *P<0.05 and ^#^*P* = 0.07.

Expression of most brown/brite markers in EP and RP+PR adipose depots at P84 was not affected by prenatal CF exposure, except there were lower transcripts of *Cited1* in EP fat of CF-offspring (Fig 4A). In IG fat, maternal CF programmed higher mRNA levels of *Ucp1* and *Cidea*, whereas *Fgf21* was lower (compared to C group). Regarding genes associated with fatty acid β-oxidation, maternal CF treatment reduced expression of *Ppargc1a* in RP+PR fat (Fig 4B). However, there were significantly higher mRNA levels of *Ppara*, *Ppargc1a* and *Cpt1b* in IG fat of CF-offspring relative to the C group.

**Fig 5:**
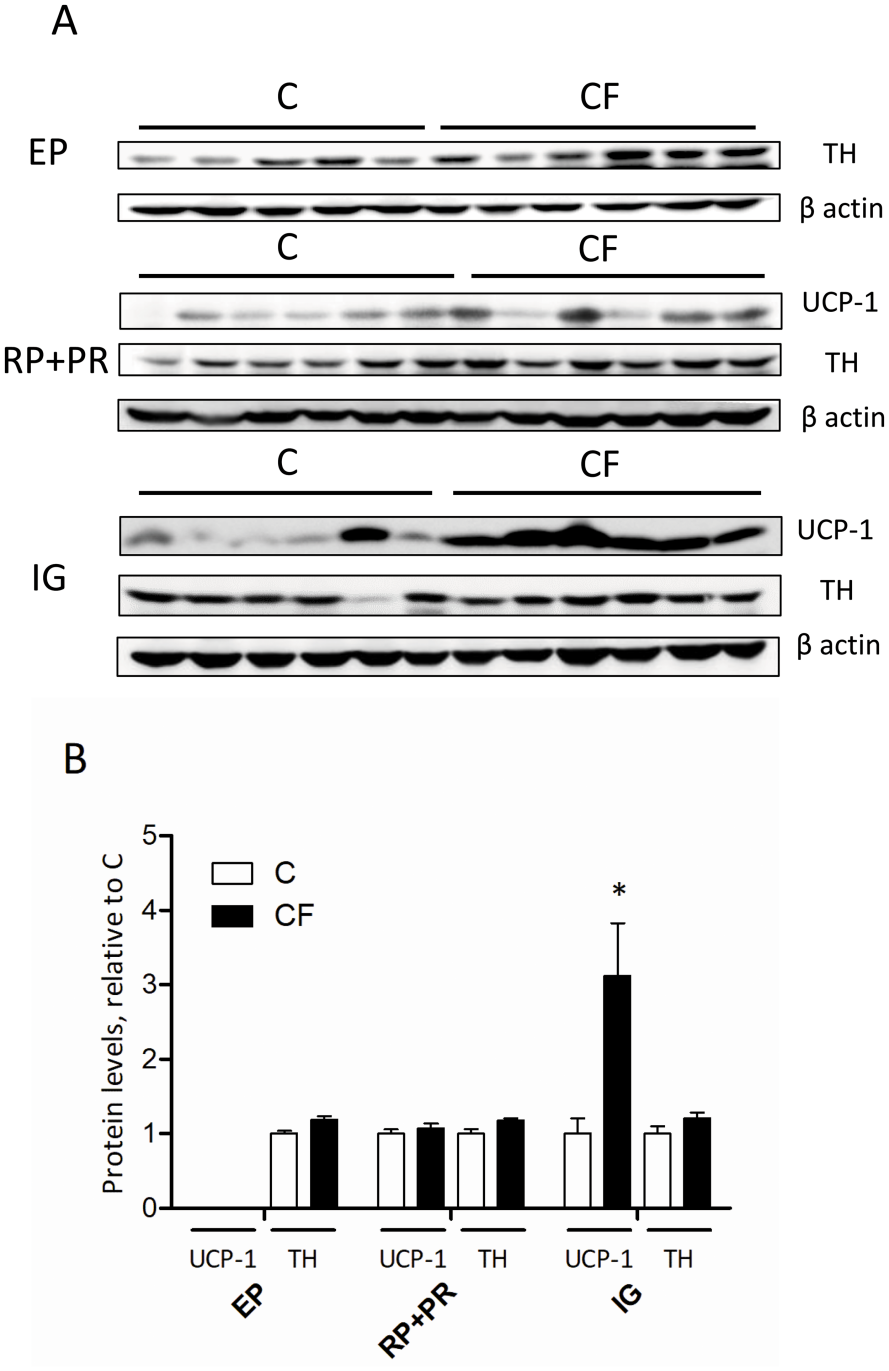
Representative picture of immunoblot (A) and protein levels of UCP-1 and tyrosine hydroxylase (TH) (B) in 3 fat depots (EP, RP+PR and IG indicates epididymal, retroperitoneal combined perirenal, and inguinal fat, respectively) of offspring at P84. Data are mean±SEM, n = 22 (from 5 litters)/group. The comparison was based on the protein levels relative to C (taking as 1) within the same fat depot. *P<0.05. In EP fat, UCP-1 is barely detectable.

To ascertain the WAT browning occurred in IG fat, protein levels of UCP1 and tyrosine hydroxylase (TH) were measured by immunoblotting in 3 fat depots at P84. In accordance with mRNA data, maternal CF administration resulted in a 3.5-fold increase of UCP1 protein amount in the IG, but not in RP+PR fat (Fig 5). In EP fat, UCP-1 is barely detectable; the detection ratio is 0 out of 22 samples in C group and 3 (with weak signals) out of 22 samples in CF group. Protein levels of TH were not different between groups in any of the 3 fat depots.

### Gestational CF on transient WAT browning of neonatal offspring

**Fig 6:**
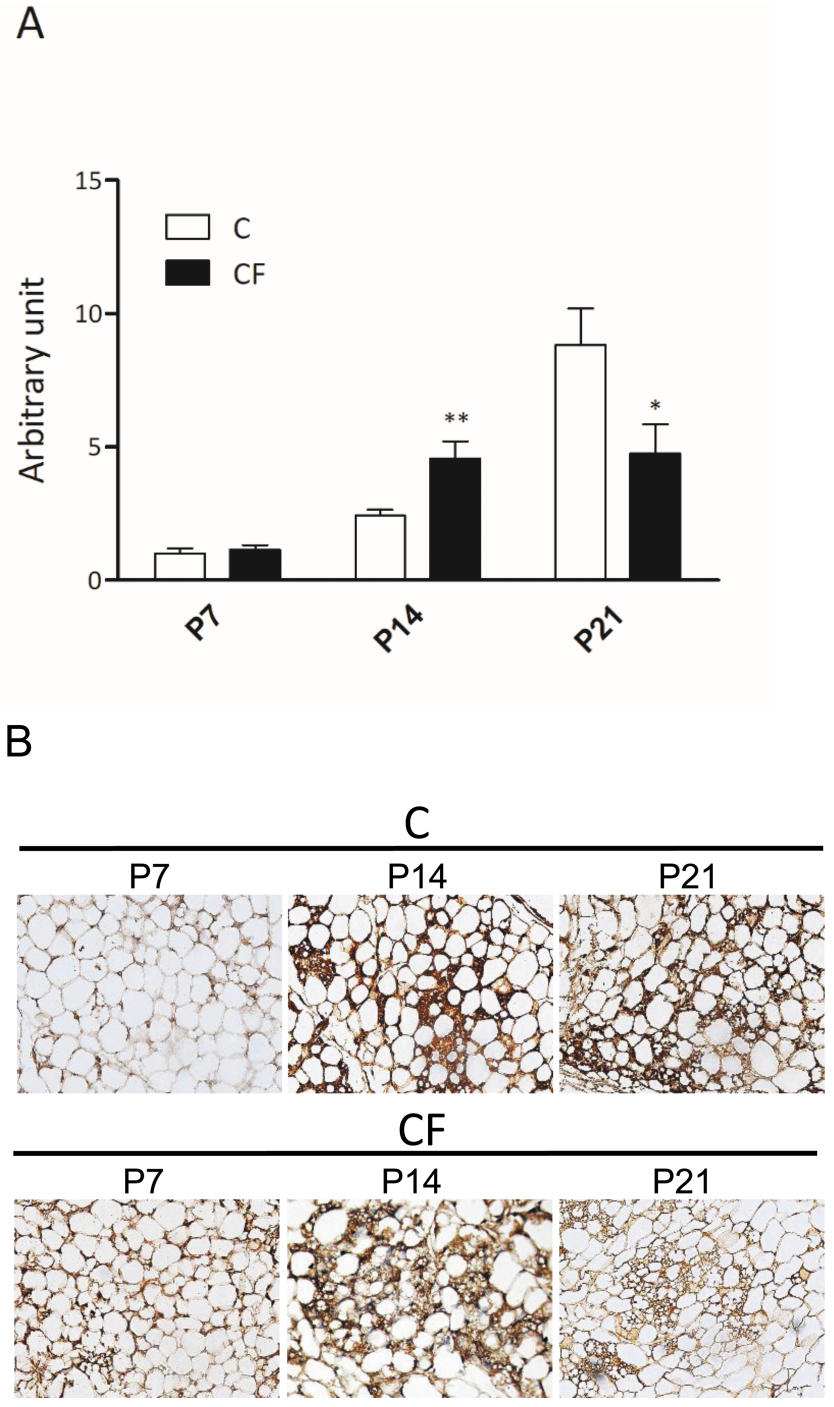
Gene expression of *Ucp1* (A) and immunohistochemical staining of UCP-1 (B) in inguinal fat of offspring during postnatal period (P7, P14 and P21). In (A), data are mean±SEM, n = 12 (from 3 litters)/group at P7, n = 10 (from 3 litters)/group at P14 and n = 14 (from 3 litters)/group at P21. The comparison was based on the mRNA levels relative to C (taking as 1 at P7) at the same time point. *P<0.05 and **P<0.01. In (B), 6 mice in each group were observed and photomicrographs shown are representatives of two groups.

Transient WAT browning was reported in the RP and IG fat around postnatal day 10–30 in rodents [31]; therefore, expression of UCP1 in the IG fat was evaluated in neonates from CF- or C-mothers at P7, P14 and P21. In C-offspring, *Ucp1* transcript in the IG had stepwise increases from P7-21 (Fig 6A). However, CF-neonates had significantly higher *Ucp1* mRNA level at P14, but significantly less at P21 than its C counterparts. Based on histochemical staining of UCP1 in IG fat, prominent WAT browning occurred at P14 for CF instead of P21 for C group (Fig 6B).

## Discussion

In this study, hepatic transcripts of *Acox* and *Cyp4a10*, two PPARα target genes were increased in CF-mothers and their fetuses at day 18 of pregnancy, then returned to basal levels (similar to their postnatal C counterparts), consistent with the duration of CF administration and the ability of CF to cross placenta [27]. Though *Fgf21* has been regarded as a PPARα target gene, its increase by CF (both hepatic mRNA levels and serum concentrations), was confirmed only in E18 fetuses but not in their mothers. Hepatic up-regulation lasted up to P7, was modest and did not affect systemic concentrations. Although CF withdrawal after birth restored PPARα activity to basal, there was a programmed effect, i.e. a greater thermogenic gene expression in subcutaneous fat of adult mice eating a HFD. This might have physiological relevance for reduced adiposity, though a prominent reduction in fat mass was not seen at present study. Maybe a longer HFD treatment is needed to reveal the difference between groups in susceptibility to obesity.

That embryonic PPARα activation caused a greater HFD-induced WAT browning in subcutaneous IG rather than in visceral EP and RP+PR WAT was ascribed to: 1) IG fat being the most thermogenic-competent tissues among WAT depots [32]; and 2) formation of primitive IG occurs before that of RP and EP. Although WAT is detectable after birth in rodents, studies directed at murine adipose tissue organogenesis have indicated subcutaneous fat begin to develop during E13.5, followed by RP is formed between perinatal period, and EP developed from P3 thereafter [23]. Accordingly, prenatal up-regulated FGF21 could act on proliferating and differentiating progenitor cells within IG and confer greater thermogenic capacity.

The increased *Ucp1*, *Cidea*, *Ppara*, *Ppargc1a* and *Cpt1b* mRNA levels confirmed greater browning capacity in IG fat of CF-adults. Garcia et al. [33] verified UCP1, CIDEA and FGF21 as good *in vivo* markers for britening. Accordingly, gene expression associated with fatty acid β-oxidation (*Ppara* and *Cpt1b*) and mitochondrial function (*Ppargc1a*) were also elevated in this study. Expression of CIDEA is high in thermogenesis-competent adipose tissues and was confirmed as an inhibitor of UCP1 activity, thereby increasing UCP1 via compensatory mechanisms [34]. Although *Tmem26*, *Cd137*, and *Tbx1* have been defined as beige adipocyte markers based on their greater expression in subcutaneous- versus BAT-derived immortalized cells lines from adrenergically treated 129 mice [2], attempts to validate these markers have produced variable outcomes. Many *in vivo* studies failed to detect their increase in WAT of mice subjected to cold stimulation [9, 33, 35]. As expression of *Tmem26*, *Cd137*, and *Tbx1* are much higher in stromal vascular fraction cells than in adipocytes, plus *Cd137* is also expressed by immune cells, the high background and potential interference from other cells might make their browning-associated induction difficult to detect, particularly in whole tissue with variable cell types [33]. However, that IG *Fgf21* was down-regulated in CF-offspring, we inferred that the increased browning capacity event was not related to FGF21 functioning in an autocrine/paracrine manner.

Although the source of brite cells has not been confirmed, we attributed the greater HFD-induced browning response in subcutaneous fat in CF-offspring to transformation between white and brown phenotypes [8, 9, 36], rather than *de novo* differentiation from progenitor brite cells [10, 37, 38]. It was reported that HFD-induced proliferation and differentiation of adipose progenitor cells occurred only in visceral, but not in subcutaneous fat [39]. In line with this note, using lineage tracing, Rosenwald et al. [9] demonstrated bi-directional interconversion between white and brite adipocytes in IG fat of mice subjected to repeated cold-warm exposure. As a consequence, former brite cells were regarded as having a memory that allowed them to become reactivated when stimulated. Perhaps the greater HFD-mediated browning response of CF-offspring was reappearance of these early brite cells induced by PPARα agonists.

Although studies in recombinant inbred mice provided strong evidence that the number of brite cells was genetically determined [40], it is still tempting to explore whether manipulation of early environment can permanently affect WAT thermogenic capacity. Recently, it was reported that maternal intervention of resveratrol throughout pregnancy and lactation induced persistent programming effects on BAT/brite function and thermogenesis in male offspring, particular in obese mothers fed a HFD [41]. Moreover, in contrast to a HFD-induced thermogenesis of subcutaneous fat to counteract excess energy intake, adult rats that were protein deficient during the perinatal period blunted this adaptation by increasing G9a, a histone methyltransferase of FGF21, thus attenuating the HFD-mediated FGF21 up-regulation and leading to an obesity-prone characteristic [42]. Perhaps an epigenetic mechanism is involved in this CF-mediated long-term effect on UCP1 induction in response to HFD. Recently, kruppel-like factor 11 (KLF11) is postulated as a browning factor to maintain (or sustain) the brite phenotype triggered by rosiglitazone (a PPARγ agonist) even the stimulus has been withdrawn in human multipotent adipose-derived stem cells [43]. KLF11 acts in a cooperative manner with PPARγ and other browning factors reprogramming PPARγ superenhancers on brite-selective genes to maintain a brite cell-specific chromatin landscape (histone marks and chromatin opening). Intriguingly, *KLF11* is not only induced by PPARγ agonist, but also has been recognized as a PPARα-regulated gene [44]. The role of KLF11 in this CF-programmed subcutaneous WAT browning will be investigated in the near future. Regardless, there may be other mechanisms (e.g. altered lipid metabolism in intrauterine environments) metabolically programming the lower adiposity and greater HFD-induced thermogenesis of these CF-exposed offspring.

In addition to cell autonomy, the role of external factors such as sympathetic innervation on this differential response between groups in WAT thermogenesis was unclear, though the mRNA levels of *Adrb3* and protein amount of TH, widely considered a marker of noradrenergic nerve fibers [45], were not increased in CF-offspring across 3 fat depots at P84. For permanent changes in sympathetic activity, rats reared at 18°C from birth to 60 d of age had a persistently greater capacity for BAT thermogenesis, mediated, in part, by more sympathetic ganglion cells innervating BAT [46]. In contrast, moderate caloric restriction during gestation in rats predisposed their offspring in future life to HFD-induced hyperplasia and fat accumulation in WAT, ascribed to reduced sympathetic innervation of IG fat [47].

In addition to working through FGF21, PPARα agonists may act directly on *Ppargc1a* (encoding PGC1α) gene to increase britening. In that regards, PPARα bound to a *Ppargc1a* promoter, an interaction potentiated by PRDM16 and synergistic with sympathetic activation, to enhance PGC1α gene transcription, consequently induces brown phenotype [20]. In this study, that prenatal CF administration increased postnatal brite cells formation was apparent at P14 (Fig 6). However, this increased UCP1 expression (relative to P7) in IG fat of CF group was lower than that of control peers at P21. A transient brown-adipocyte phenotype (BAP) in developing IG and RP fats has been reported in mice from postnatal days 10–30, although involution occurred at approximately 35–56 d of age, but subsequently re-appeared after stimulation [31]. In accordance with the adult susceptibility to DIO and response to β-adrenergic agonists or cold, A/J and 129 mice had much greater magnitude of this postnatal transient BAP as compared to obesity-prone C57BL/6 mice [11, 12, 48]. The physiological relevance of this postnatal transient BAP remains elucidated, since the time point of its occurrence does not coincide with a postnatal thermogenic requirement. Furthermore, diets of these young pups have switched from high fat (milk) to high carbohydrate at this time point. Moreover, the relevance of postnatal BAP to future thermogenic capacity of WAT is ambiguous. Using lactational undernutrition or rearing at 17°C (vs. 29°C) during lactation to suppress and enhance, respectively, postnatal BAP, Kozak et al. reported no influence on either DIO- or cold-induced thermogenesis in later life, though both strategies conferred increased resistance to DIO [49, 50].

From the UCP1 expression detected at P7, P14 and P21, we concluded that the time span of postnatal BAP was hastened by prenatal PPARα activation. As postnatal changes in hepatic FGF21 expression preceded the wax and wane of WAT UCP1 and 129 mice always has a higher FGF21 levels than C57BL/6, Lasar et al. [48] inferred FGF21 was one factor controlling postnatal BAP. The current results supported this view, as embryonic FGF21 induction led to an early-onset BAP. In future studies, we will determine how this time shift affects later responses of UCP-1 induction.

Oxidized modified fatty acids were potent PPARα agonists in frying oils; however, this is not recommended for consumption during pregnancy, as abused frying oil may be teratogenic due to disturbed retinoic acid metabolism [26]. In addition to pharmacological agents, there are many PPARα agonists in natural food materials that lack the adverse side effects of frying oil. Although underlying mechanisms are not clearly elucidated, this study provided proof of concept that mothers may confer their offspring greater thermogenic capacity of WAT by consuming appropriate dietary components during pregnancy.

## Conclusion

Giving CF to pregnant mice increased hepatic mRNA and circulation levels of FGF21 in fetus. This prenatal CF exposure hastened postnatal transient brown-adipocyte phenotype and programmed a greater HFD-induced thermogenic gene expression in subcutaneous, but not in visceral WAT, in male offspring.

## Supporting information

S1 Table Compositions of the test diets used in this study. (PDF)

S2 Table Assay ID of the inventory primers and probes and the sequence of the self-designed primers used for qRT-PCR. (PDF)

## Abbreviations

BAP: brown-adipocyte phenotype
BAT: brown adipose tissue
C: control
CF: clofibrate
DIO: diet-induced obesity
EP: epididymal
FGF21: fibroblast growth factor 21
HFD: high-fat diet
IG: inguinal
KLF11: kruppel-like factor 11
PR: perirenal
RP: retroperitoneal
TH: tyrosine hydroxylase
WAT: white adipose tissue
