# Supplementary material for: Prenatal PPARα activation by clofibrate increases subcutaneous fat browning in male C57BL/6J mice fed a high-fat diet during adulthood
Source: PLoS One. 2017 Nov 2;12(11):e0187507. doi: 10.1371/journal.pone.0187507 (PMC5667850; doi:10.1371/journal.pone.0187507)
Supplement: S1 Table — (PDF) [file pone.0187507.s001.pdf]

**Supporting Table 1.** Compositions of the test diets used in this study

| Ingredients | C | CF | HFD |
|---|---|---|---|
| | g / kg diet | | |
| Corn starch | 561 | 561 | 160 |
| Sucrose | 100 | 100 | 160 |
| Casein | 140 | 140 | 260 |
| Clofibrate[1] | - | 5 | - |
| Fresh soybean oil | 100 | 100 | 10 |
| Butter oil | - | - | 290 |
| Fiber | 50 | 50 | 61 |
| Mineral mixture (AIN-93) | 35 | 35 | 42 |
| Vitamin mixture (AIN-93 M) | 10 | 10 | 12 |
| L-Cystine | 1.8 | 1.8 | 3 |
| Choline bitartrate | 2.5 | 2.5 | 2 |
| tert-Butylhydroquinone | 0.008 | 0.008 | |

[1] >98.0% purity Clofibrate (Fluka)
