# Supplementary material for: Prenatal PPARα activation by clofibrate increases subcutaneous fat browning in male C57BL/6J mice fed a high-fat diet during adulthood
Source: PLoS One. 2017 Nov 2;12(11):e0187507. doi: 10.1371/journal.pone.0187507 (PMC5667850; doi:10.1371/journal.pone.0187507)
Supplement: S2 Table — (PDF) [file pone.0187507.s002.pdf]

Supporting Table 2. Assay ID of the inventory primers and probes and the sequence of the self-designed primers used for qRT-PCR

Gene	Accession number	Assay ID or primer sequence
Ucp1	NM_009463.3	Mm01244861_m1
Fgf21	NM_020013.4	Mm00840165_g1
Pparα	NM_011144.6	F: CAGCAACAACCCGCCTTTT<br>R: GCAGTGGAAGAATCGGACCTC
Acox1	NM_015729	F: CCAAGATTCAAGACAGAGCC<br>R: TCCCCTCAAGAAAATCCCC
Cyp4a10	NM_010011.3	F: TGAGGGAGAGCTGGAAAAGA<br>R: CTGTTGGTGATCAGGGTGTG
Cidea	NM_007702.2	F: AGGGACAGAAATGGACACCGGGTAG<br>R: GCCGAGGAAGTCCTTGGGGTT
Cited1	NC_000086.7	F: AACCTTGGAGTGAAGGATCGC<br>R: GTAGGAGAGCCTATTGGAGATGT
Cd137	NM_001077509.1	F: CGTGCAGAACTCCTGTGATAAC<br>R: GTCCACCTATGCTGGAGAAGG
Tmem26	NM_177794.3	F: ACCCTGTCATCCCACAGAG<br>R: TGTTTGGTGGAGTCCTAAGGTC
Tbx1	NM_011532.1	F: GGCAGGCAGACGAATGTTC<br>R: TTGTCATCTACGGGCACAAAG
Ppargc1a	NM_008904	F: GTAGGCCCAGGTACGACAGC<br>R: CTCTTTGCGGTATTCATCCC
Cpt-1b	NM_009948.2	F: TGCCTTTACATCGTCTCCAA<br>R: GGCTCCAGGGTTCAGAAAGT
Adrb3	NM_013462.3	F: GCAGGAGGAAGATGGAAACCA<br>R: GAACCTGAGGCAACCCCTCT

[1] Inventory primers and probes purchased from Applied Biosystems
